# The impact of COVID-19 on safe abortion access in Africa: An analysis through a framework of reproductive justice and lens of structural violence

**DOI:** 10.3389/fgwh.2022.958710

**Published:** 2022-09-23

**Authors:** Amanda Tiew, Lucía Berro Pizzarossa, Ibtehal Jastaniah, Ruvani T. Jayaweera

**Affiliations:** ^1^The International Network for the Reduction of Abortion Discrimination and Stigma (inroads), Seattle, WA, United States; ^2^O'Neill Institute for National and Global Health Law, Georgetown University, Washington, DC, United States; ^3^Ibis Reproductive Health, Cambridge, MA, United States; ^4^Ibis Reproductive Health, Oakland, CA, United States

**Keywords:** COVID-19, abortion, Sub-Saharan Africa, reproductive justice, structural violence, self-managed abortion

## Abstract

The SARS-CoV-2 virus causing the coronavirus disease (COVID-19) global pandemic heightened restrictions on sexual and reproductive health and rights (SRHR), especially concerning safe abortion access. The African region has been particularly susceptible to the impact of COVID-19 on sexual and reproductive health services. Using a framework of reproductive justice, we interviewed key informants from the Mobilizing Action around Medication Abortion (MAMA) Network regarding the impacts of structural violence and COVID-19 on SRHR programming in Africa, particularly programming on self-managed abortion. We identified themes of lacking infrastructures of support, emergent marginality, and neocolonial funding environments as facets of structural violence within the context of the MAMA Network, as heightened by the COVID-19 global pandemic.

## Introduction

The SARS-CoV-2 virus causing the coronavirus disease (COVID-19) pandemic and resulting lockdowns greatly impacted sexual and reproductive health and rights (SRHR) in Africa, as local and national resources were strained by long-term structural issues such as supply-chain disruptions and de-prioritization of SRHR services (1–3). The pandemic aggravated an existing “silent pandemic” on the African continent: the highest proportion of unsafe abortions in the world (4–7).

In this challenging landscape for abortion access, self-managed medication abortion (SMA) and the crucial work of grassroots activists hold particular promise (4). The Mobilizing Activists around Medical Abortion (MAMA) Network, founded in 2016, is a collaboration of 54 organizations of grassroots activists and feminist groups working within 22 countries of the African region to expand access to safe abortion (SA) through SMA (8), which research has demonstrated is safe and effective (9–13). Indeed, the World Health Organization recently updated their abortion guidance to include self-managed medication abortion as a recommended method of abortion (14). Through evidence-based and stigma-free information sharing, the MAMA Network engages a wide range of strategies to enable safe self-management trajectories, such as hotlines, community outreach, and technological innovations (8). Their work remains crucial to the African regional context. Though the legal status of abortion and political and economic circumstances differ across the many countries the MAMA Network operates, tapping into the potential of SMA can radically transform the abortion landscape in all country contexts, curbing maternal mortality and morbidity rates and enhancing autonomy (15, 16).

Nandagiri et al. describe how “institutionalized and everyday forms of violence restrict and affect abortion access,” ranging from racism in healthcare to policies banning SMA (17). They posit that a structural violence lens allows us to move away from the individualization of reproductive decision-making, which often locates the burden of accessing SA care upon the individual pregnant person (17, 18). Chiweshe et al. defines reproductive justice as locating reproduction within the social power relations of a particular context. They reject the more commonly touted “reproductive choice” framework as Western-centric and less relevant to African contexts. Locating the research from a framework of reproductive justice repositions reproductive rights in a sociopolitical context of intersectionality, including race, gender, sexuality, class oppressions, and more (19).

Using these frameworks, we explored the ways in which COVID-19 exacerbated existing structural violence and further impacted the ability of MAMA members to uphold abortion access (20). To understand the impact of COVID-19 on members, we conducted virtual key informant interviews with 15 member organizations from 11 countries between October and November 2020 using a semi-structured interview guide. All interviews were conducted individually in English or French, and recorded, transcribed, and translated to English. Three interviewers were responsible for conducting the interviews. We use these quotes in this perspective to highlight how the pandemic emphasized the lack of supportive infrastructure, the emergent marginality of MAMA Network organization beneficiaries, and international funder and policy influences.

## Unveiling the lack of supportive infrastructures

A lack of supportive infrastructures, which refers to the basic foundational services and systems that offer the necessary support to enable day-to-day operations and workflow, is a form of structural violence. Within the context of SA access in Africa, gaps in transportation, communications, data security infrastructures, and infrastructures of care posed multi-layered challenges for members of the MAMA Network and their beneficiaries.

Impacts on beneficiaries related to how gaps in transportation affected overall costs. As governments shut down public transportation services, beneficiaries had to rely on private transportation to access services throughout their abortion trajectories, increasing their overall costs (20). MAMA members reflected on the costs of abortion as holistic, factoring in transportation costs when appraising overall abortion costs. Other sustained costs include time, interruption of care responsibilities, and loss of pay for those in the informal economy.

“[Beneficiaries] say access to SA is very expensive, because they have to move one place to another and some move a long distance, so they say it is very expensive…that is why maybe a lot of people [have] an unsafe abortion in their areas...Because they need transportation, food, to pay for the services, so they just combine all of these and say it's expensive now.”

From an organizational perspective, most organizations were impacted by a lack of portable computers, smartphones, stable internet access, and network connectivity in rural areas during the lockdown period. Without access to necessary equipment, staff experienced decreased productivity while working from home during lockdown measures. In-person programmes were also affected by the transition to virtual programming, as participants lacked access due to the same connectivity and equipment needs. Some organizations responded with innovative solutions, such as buying monthly internet passes for Community Health Workers (CHWs) and distributing laptops to encourage a socially distanced approach to attending their online programmes.

The shift to virtual workspaces increased the need for digital security measures, particularly in the context of internet crackdowns and the criminalization of activists and people who self-manage their abortions (21). Without necessary digital security infrastructure during this transition, organizations expressed concerns of digital and data insecurity, which had previously been mitigated by in-person meetings and familiar office settings.

“We were worried about having intruders in the meetings when we're talking about issues on access to SMA… If something happens to these people through the meetings, how can the organization be protected?”

Employees of MAMA organizations, all cisgender women, also expressed difficulties balancing professional employment with their domestic roles due to absent infrastructures of care. These holistic systems of support, such as childcare and elderly care, often help to support the wellbeing of others and their caretakers, namely, working women who hold simultaneous responsibilities of economic provision and feminized care (22–24). Without these supportive infrastructures during the lockdown period, the double-burden of domestic and professional roles proved difficult to maintain:

“We were affected because when you're working from home, you have other things. Children are around, schools were closed, and being an effective worker was not very easy.”

The strain of these roles was amplified by the sensitive and urgent nature of their work in SA care, being on the frontlines of service provision and having to navigate lockdown and the resulting unmet needs of beneficiaries. This also brought upon requests for mental health support amongst employees, as seen here:

“We asked that we consider incorporating mental health into our work too, because some of our staff [were] not coping with work from home.”

As indicated, staff members began requesting mental health support to cope with a lack of infrastructural support, exacerbated by the pandemic, signaling a need that might not have been new, but had reached a point of requiring attention. This can be inferred to have compounded from the increased burden of feminized care that employees and governments had not been structurally prepared to support (25).

In terms of pathways to MA access, many MAMA organizations held and continue to hold longstanding partnerships with community pharmacists, who agreed to serve as a reliable source of MA pills to beneficiaries in need. However, the pandemic underscored new barriers of access within these supportive infrastructures (20).

“All [the pharmacists] are thinking of is COVID-19. They can't even listen to us. When we are trying to get in touch with them, they are like, we can't do that right now.”

With national lockdowns and border closures, access to MA pills became scarce and expensive without infrastructures to support access (3, 20). Structural violence is embedded in the compounding difficulties of accessing MA pills, where those who had been vulnerable prior to the pandemic became additionally so, resorting to extremes in attempting to access abortion:

“One of [the beneficiaries] even told us that since the pills were very scarce, they could split the pills, like one pill could serve two girls, which is so dangerous...The scarcity of the pills was the big challenge on women accessing SA services.”

Countries around the world have noted similar pressures on their healthcare systems, leaving frontline healthcare workers overwhelmed by the burdensome decisions of prioritizing the most urgent healthcare needs of the nation (26). In Africa, where healthcare infrastructures lack support and funding, this conundrum occurred at the direct expense of SA provision (20, 27).

## Emergent marginality

Marginality is defined by Gatzweiler as: “An involuntary position and condition of an individual or group at the margins of social, political, and economic systems, that prevents them from access to resources, services, freedom of choice” (28). Emergent marginality is marginality that has always existed, yet emerges to become suddenly visible during circumstances of crisis, such as the COVID-19 lockdown (26). Within the context of the MAMA Network, the marginalities that became apparent during the pandemic included the effects of economic need, barriers to medication abortion (MA) access, and gender-based violence (GBV).

As lockdown progressed, organizations shifted to address the ever-changing needs of beneficiaries, with funds reallocated toward direct food and emergency aid. Some organizations, bound by donor funding commitments, found innovative ways to provide emergency aid to beneficiaries, such as leveraging partnerships to provide needed supplies:

“With the support of different partners, some beneficiaries were provided with food supplies for them to survive.”

The word “survive” suggests the dire situation of community members during the pandemic. One organization mentioned providing essential items such as underwear and menstrual health products for girls and women taken in for domestic violence shelter, alongside the organization's regular provisions of SRHR-related aid:

“We had to make emergency set lists for them. They need clothing, underwear, everything…We provided those things for them.”

These rapidly shifting needs resulted from unemployment and economic needs exacerbated by the lockdown period (29, 30). Although organizations within the MAMA Network share a mission of addressing SA access and SRHR-related topics, organizations mobilized to provide for the most urgent needs of their beneficiaries as well.

GBV, the “shadow pandemic”, presented itself throughout the emergency aid work and lived experience of MAMA Network organizations as yet another glaring emergent marginality during the COVID-19 pandemic (31). Lockdowns kept women and girls at home, without protection from the same perpetrators of violence that they often lived with (20). A projection in 2020 predicted that for every 3 months of lockdown, an additional 15 million cases of GBV were expected in Africa (32).

Organizations noted the need to redirect their SA and reproductive health hotlines toward addressing GBV during the pandemic, in response to the heightened levels within communities:

“We had high rates of GBV with limited to no support during COVID-19…We expanded [our] priorities to accommodate COVID-19 challenges of GBV and began to offer free counseling and information.”

These quotes highlight the specific and unique circumstances magnified by lockdown restrictions, where women with violent partners found themselves suddenly isolated from the people and resources that might support them (32).

## Neocolonial foreign influence

Global health financing enacts structural violence through its long-embedded, well-accepted structures of international aid, donor financing, and donor approval (33). By determining which services can or cannot be provided, global health financing imposes individual, organization-level, or national policy interests through a Western-centric lens, without considering the knowledge, needs, and contexts of on-the-grounds organizations (34). The Mexico City policy, first implemented in 1985, exists as the most visible example of Western-centric neocolonial foreign influence, and is widely documented to have interfered with international efforts to provide access to SA services (35, 36). Its enactment contributes to long-term impacts on organizations which require stable programmatic development in order to achieve long-term change within their own countries (37). This becomes difficult when organizations are reliant on international funding that are influenced by foreign agendas outside of their own vision for increasing SA access (34). Given this reliance, the Mexico City policy, atop significant cuts in GBV and SRHR global funding during the pandemic, resulted in great impacts on MAMA organizations' operations and reach (38).

At least one organization noted the influence of the Mexico City policy and how their working partners reported barriers to service provision due to the threat of funding withdrawal:

“They told us they had the challenge of the Mexico City Policy where some of the services we wanted from them, they were not in a position to provide.”

In addition to navigating barriers posed by the Mexico City policy, organizations also experienced a loss of funding from donors during the pandemic (38). This left them unable to continue SRHR and abortion programming:

“Most of our donors have stopped funding our organization...We are suffering a lot because we don't have funding to sustain our programs.”

Lack of donor support during the pandemic did not manifest only through limited funding, but also through a lack of flexibility in programs and increased costs, as evidenced in this quote:

“COVID also affects our programming in terms of the budget, because we have to purchase for every participant hand sanitizers, face masks, soap, and water and we have to maintain social distancing. There are all those things that have come in that we did not program at the beginning as we were planning for our financial year... Our programming has been impacted quite hard.”

## Discussion

African researchers such as Malvern Chiweshe have rejected the Western-centric framework of “reproductive choice” in discussing abortion access, noting its lack of relevance and accessibility within African sociocultural and sociopolitical settings (19). “*Choice, it was argued, is the preserve of the privileged”* (39). Instead, Chiweshe and other African feminist researchers have supported using the framework of reproductive justice—coined by Black women activists in the US to encompass human rights, reproductive health, and social and racial justice—to seek and understand how health inequalities may be eradicated in Africa through processes of decolonization and an interrogation of structural inequalities (19, 39–41). Through a reproductive justice framework, abortion is linked to African “women and girls' economic, social, and political power and resources to make healthy decisions about their bodies, sexuality, and reproduction for themselves, their families, and their communities in all areas of their lives” (19).

It is through Chiweshe's call for decolonization that the lens of structural violence allowed us to critically examine the experiences of the MAMA Network during the first year of pandemic lockdowns (17, 19). Across the MAMA Network, there was a prominent, compounded effect of a lack of supportive infrastructures and emergent marginality of beneficiaries. Without governmental support to ensure adequate assistance and infrastructural support during a time of crisis, many countries saw a direct increase in unsafe abortions performed within these communities during the pandemic (1, 42). The compounded impact of COVID-19 upon the most vulnerable in society is a direct reflection of the structural violence enacted in the absence of supportive infrastructures and advocacy.

In the same line, this perspective clearly demonstrates the importance of support from CHWs who live amongst and work with the communities they serve. As Ngozi Erondu writes, many countries facing endemic diseases and poor health systems rely heavily on CHWs that are closest to the ground (33). As a network largely staffed by CHWs, MAMA organizations operate at the heart of their constituencies, possessing homegrown intimacy and abilities to understand their beneficiaries' healthcare needs and barriers, particularly when it came to SA access during lockdown (8).

Perhaps most striking, amidst the conflux of issues faced in providing SA access during the pandemic, was the magnifying lens COVID-19 held to organizations' reliance on international funding. Criticisms of donor-reliant funding include how it places the delivery of health interventions into the hands of Western NGOs, creating a neocolonial global health financing model which diminishes the autonomy of local programs to deliver to their populations, ultimately leading toward weaker health delivery systems overall (33). Organizations reflected this in their struggles accessing urgently needed tools, such as laptops and smartphones, whilst managing influxes of demand on their hotlines. Instead, working capacity that was already lacking amongst staff had to be redirected toward requesting funding and/or seeking additional donors during the COVID-19 crisis (43).

Legal restrictions through international policy interference, as exemplified in the Mexico City policy, made access to SA harder (44). As a result, some organizations were forced to focus on post-abortion care to avoid being penalized for work amongst their own constituencies. Thus, a model of heavy reliance on global health financing can prove precarious, as this reliance is predicated on requirements which do not always align with community needs, creating new forms of neocolonial control in LMICs, rather than decolonizing global health (34).

In the face of the pandemic and various degrees of structural violence, MAMA organizations remained flexible and present in responding to ever-changing needs and challenges on a grassroots level. It is precisely this persistence, coupled with a call to decolonize global health and reproductive justice, that inspires equitable access to SA care for communities in the face of COVID-19 and structural violence (17).

## Implications for policy on abortion in Africa

Our findings have important implications for abortion policy in Africa. Firstly, our research highlights the crucial role played by grassroots activists as part of the constellation of actors that work locally and transnationally, enabling safer abortion trajectories while also providing different types of support (41). In terms of SMA, our findings align with recently adopted WHO guidelines that embrace the potential of self-managed abortion, calling for the decriminalization of those who self-manage and those who are doing the crucial work of supporting safe(r) trajectories (14). While systemic changes regarding health systems can be complex, COVID-19 highlighted the potential for these countries to achieve self-sufficiency and empowerment through their healthcare systems, separate from reliance on global health financing, by utilizing the existing networks and connections of CHWs (45). Training programmes and policies to continue empowering national healthcare systems with CHWs in the long-term should be developed, as well as long-term solutions to fund their efforts and to create motivation within communities for these roles (46).

Lastly, it is undeniable that governments hold a significant responsibility in providing the supportive infrastructures, such as public transportation, regular and constant electricity, MA pill provision in public hospitals and pharmacies, and subsidized childcare. These are significant issues which must be supported and given allocation within national budgets, as well as advocated for by civil service organizations. However, political histories and neocolonial enmeshments in Africa must also be considered within these recommendations (34). International commitments to the beleaguered health systems of the Global South, particularly in Africa, such as the Paris Declaration on Aid Effectiveness and the Dakar Agenda for Action call for implementing sustainable models of global health financing, with the long-term mission to empower autonomy and achieve donor-independence (47). Calls for equity-driven funding through the mission of decolonizing global health and global health financing can then go one step further toward justice, more than equity, within funding efforts (20, 48).

## Data availability statement

The original contributions presented in the study are included in the article/supplementary materials, further inquiries can be directed to the corresponding author.

## Author contributions

AT contributed significantly with conception, analysis, and writing. AT, LB, IJ, and RJ contributed significantly to editing the perspective. All authors contributed to the article and approved the submitted version.

## Funding

Funding for this work was supported by Amplify Change and the Safe Abortion Access Fund.

## Conflict of interest

The authors declare that the research was conducted in the absence of any commercial or financial relationships that could be construed as a potential conflict of interest.

## Publisher's note

All claims expressed in this article are solely those of the authors and do not necessarily represent those of their affiliated organizations, or those of the publisher, the editors and the reviewers. Any product that may be evaluated in this article, or claim that may be made by its manufacturer, is not guaranteed or endorsed by the publisher.
